# Skin diseases in pediatric patients attending a tertiary dermatology hospital in Northern Tanzania: a cross-sectional study

**DOI:** 10.1186/s12895-015-0035-9

**Published:** 2015-09-10

**Authors:** Samson K. Kiprono, Julia W. Muchunu, John E. Masenga

**Affiliations:** Department of Dermatology, Regional Dermatology Training Center, P.O. Box 8332, Moshi, Tanzania; Department of Medicine, Moi University School of Medicine, P.O. Box 4606–30100, Eldoret, Kenya

**Keywords:** Pediatric, Skin diseases, Africa

## Abstract

**Background:**

Skin diseases affect 21–87 % of children in developing countries in Africa. However, the spectrum of the skin diseases varies from region to region due to several factors such as genetics, socioeconomic and environmental. The aim of this study was to determine the spectrum of childhood skin diseases in Tanzania.

**Methods:**

We conducted a prospective hospital- based cross-sectional study between September 2012 and August 2013 at a tertiary referral dermatology clinic. Children younger than 14 years presenting with new skin conditions were recruited. Diagnosis was mainly done clinically, but if the diagnosis was not clinically clear, further investigations were undertaken accordingly.

**Results:**

A total of 340 patients were recruited of which 56 (16.5 %) had more than one skin condition. Both genders were equally affected. Infections and infestations accounted for the majority (43.5 %, *n* = 177) of the skin conditions followed by eczematous dermatitis (28.5 %, *n* = 116) and pigmentary disorders (7.4 %, *n* = 30). Among the 152 infectious skin diseases, fungal infections predominated (50.7 %, *n* = 77) in the infectious group followed by bacterial (29.6 %, *n* = 45), and viral (19.7 %, *n* = 30).

**Conclusions:**

Skin infections are still the main cause of dermatological consultations in children although with a reduced prevalence. Inflammatory skin conditions are increasing and can be attributed to improved socioeconomic status and HIV pandemic.

## Background

Skin diseases affect 21–87 % of children in African developing countries and constitute up to a third of outpatient visits to Pediatricians and Dermatologists [1, 2]. Despite their common occurrence, skin diseases receive less attention as compared with diseases such as malaria, pneumonia and HIV/AIDS, which cause significant mortality [3].

The spectrum of skin diseases differs in different parts of the world. The patterns of skin diseases have been shown to vary according to environmental and socioeconomic factors [2]. Eczema has been reported to be the predominant skin disease in developed countries, whereas infections and infestations are predominant in developing countries [2, 4]. Infections account for 40–80 % of all skin diseases in sub-Saharan Africa [5], and most of these diseases are preventable. However, Dlova et al. [5] reported changing trends in skin conditions among black South Africans with an increase in inflammatory conditions. In a community based study done in the 1990s in Tanzania, it was reported that one-half of the participants with skin diseases were children younger than 15 years and the majority had infections [6]. Sub-Saharan Africa has realized socioeconomic changes over the last 2 decades. Similarly during the same period there was an increase in the number of people living with HIV due to availability of anti-retroviral drugs. Therefore the aim of this study was to determine the current spectrum of childhood skin diseases in northern Tanzania.

## Methods

This was a cross-sectional study conducted at The Regional Dermatology Training Center (RDTC) skin clinic, which is a tertiary referral clinic in Northern Tanzania. All children younger than 14 years who came to the clinic with new skin disorders between September 2012 and August 2013 were randomly recruited by selecting every third patient that was registered in the clinic until the desired calculated sample size of 340 was obtained. The diagnosis was mainly done clinically, but relevant laboratory investigations or histopathology was done in cases with unclear diagnosis. Outcomes were analyzed with SPSS version 16 and summary statistics obtained. Ethical approval for the study was granted by The Kilimanjaro Christian Medical University College ethics and research board.

## Results

**Fig. 1:**
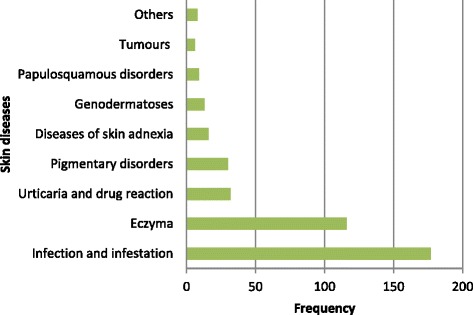
The spectrum of skin diseases in 340 children treated at the Regional Dermatology Training Center

A Sample 340 children out of 1,339 children were sampled with a male to female ratio of 1:1. The median age was 4.2 years and ranged from 1 week to 13.9 years. The majority (60 %) of the children resided in urban areas. A total of 407 skin diseases were diagnosed in 340 children with 56 (16.5 %) children having more than one skin condition. Skin infections and infestation (43.5 %, *n* = 177) were the most common group of skin diseases. Other common groups shown in Fig. 1 are eczema (28.5 %, *n* = 116) and Pigmentary disorders (7.4 %, *n* = 30). Six (1.5 %) children were diagnosed with tumors. The skin diseases were more common in children under the age of 5 years (*n* = 179, 52.6 %) while (*n* = 87, 25.6 %) and (*n* = 74, 21.8 %) was seen in age group of 5–10 years and above 10 years respectively.

The distribution of the skin diseases among 340 children are shown on Table 1. Among 152 children diagnosed with skin infections, 50.7 % (*n* = 77) had fungal infections consisting mainly of tinea capitis (84.4 %, *n* = 65). Impetigo was the commonest (46.7 %, *n* = 21) diagnosis among the 45 children with bacterial infections. Other common bacterial infections were furuncles (24.4 %), folliculitis (15.6 %) and ecthyma (13.3 %). Scabies was the only infestation which was diagnosed in 25 patients.

**Table 1:** Frequency of 407 cutaneous diseases seen in 340 children attending tertiary dermatology clinic

Diseases	Frequency (%)
Bacterial infections (*n* = 45, 13.2 %)	
Impetigo	21 (6.2)
Furuncles	11 (3.2)
Folliculitis	7 (2.1)
Ecthyma	6 (1.8)
Fungal infections (*n* = 77, 22.6 %)	
Tinea capitis	65 (19.1)
Tinea facei	4 (1.2)
Tinea corporis	7 (2.1)
Tinea cruris	1 (0.3)
Viral infections (*n* = 30, 8.8 %)	
Warts	15 (4.4)
Molluscum contagiosum	5 (1.5)
Herpes simplex	4 (1.2)
Varicella	4 (1.2)
Palmoplantar warts	1 (0.3)
Measles	1 (0.3)
Infestations (*n* = 25, 7.4 %)	
Scabies	25 (7.4)
Eczematous dermatitis (*n* = 116, 34.1 %)	
Atopic dermatitis	107 (31.5)
Contact dermatitis	5 (1.5)
Seborrheic dermatitis	3 (0.9)
Nummular eczema	1 (0.3)
Papulosquamous disorders (*n* = 9, 2.6 %)	
Pityriasis rosea	7 (2.1)
Psoriasis	1 (0.3)
Lichen planus	1 (0.3)
Urticaria and drug reaction (*n* = 32, 9.4 %)	
Papular urticarial	19 (5.6)
Papular pruritic eruption	5 (1.5)
Urticaria and angioedema	4 (1.2)
Fixed drug reaction	3 (0.9)
Drug exanthema	1 (0.3)
Pigmentary disorders (*n* = 30, 8.8 %)	
Vitiligo	10 (2.9)
Pityriasis alba	10 (2.9)
Nevi	7 (2.1)
Postinflammatory hyperpigmentation	2 (0.6)
Postinflammatory hypopigmentation	1 (0.3)
Disorders of skin adnexa (*n* = 16, 4.7 %)	
Acne	11 (3.2)
Miliaria	5 (1.5)
Genodermatosis (*n* = 13, 3.8 %)	
Albinism	7 (2.1)
Xeroderma pigmentosa	3 (0.9)
Itchyosis	2 (0.6)
Neurofibromatosis	1 (0.3)
Tumors (*n* = 6, 1.8 %)	
Kaposis sarcoma	2 (0.6)
Basal cell carcinoma	1 (0.3)
Hemangiomas	3 (0.6)
Others (*n* = 8, 2.4 %)	
Keloid	5 (1.5)
Xerosis	3 (0.9)

Among 230 non-infectious skin diseases seen in this study, the majority (50.7 %, *n* = 116) was in the eczematous dermatitis group. Atopic dermatitis (92.2 %, *n* = 107) was diagnosed in the majority of these patients with eczema. Pigmentary disorders (13.1 %, *n* = 30), urticaria and drug reaction (13.3 % *n* = 32) and genodermatosis (5.7 %, *n* = 13) were among the top five non-infectious dermatoses.

HIV test was done in 243 (71.5 %) children older than 18 months. The HIV prevalence rate in this group was 5.8 % (*n* = 14). The median CD4 count was 636cells/mm3 with an interquartile range of 279.25. The mean age of the HIV positive children was 8.4 years. Six patients were currently on anti-retroviral treatment. Skin diseases seen in the 14 HIV positive children were flat warts (28.6 %, *n* = 4), papular pruritic eruption (28.6 %, *n* = 4), tinea capitis (21.4 %, *n* = 3), Kaposi sarcoma (14.3 %, *n* = 2) and seborrheic dermatitis (14.3 %, *n* = 2). There was no association (*p* = 0.438) between HIV status and cutaneous infections.

## Discussion

This study was conducted at a tertiary referral skin clinic. The findings may not be generalized to other hospitals in the region or represent the true spectrum of the diseases in the community.

Skin diseases are still a major cause of morbidity in children in sub-Saharan Africa [1, 2]. Majority of the skin diseases occur in children under the age of 5 years. This high prevalence could be due to the lower immunity or higher frequency of hospital visits by infants due to greater parental care. Skin infections are the most predominant skin diseases in children in this study similar to others in developing countries [3, 4] but in contrast to those reported in developed countries [1, 7]. The high prevalence of infections and infestations in the African developing countries has been attributed to low socioeconomic status, favorable tropical weather, neglect and poor hygiene [6, 8]. Fungal infections and especially tinea capitis are the most prevalent infections in all ages.

This could be due to sharing of shaving machines which a common practice in the community.

The low prevalence of viral infections is in contrast to other studies which have shown cutaneous warts to be the most common infective dermatosis [9]. This may be due to environmental factors, HIV co-infection or difference in level of resistance to human papillomas virus among ethnic communities. Few cases of infestations (all scabies) were diagnosed in this study in contrast to what is expected to be in the community. Community based studies done in Tanzania towards the end of last century showed the prevalence of transmissible diseases to be as high as 84 % [6, 10–12] while the current study shows that infections and infestations are still the most common group of skin diseases seen in a tertiary hospital albeit with a lower prevalence.

According to Gibbs [6], poor socioeconomic status was the only significant factor associated with transmissible diseases. An improvement in social and economic situation during the last 2 decades in Africa may have contributed to a lower prevalence of infectious diseases. Henderson [12] reported the prevalence of Tungiasis and Pediculosis to be 1.5 and 5 % respectively in rural parts of Tanzania. However in this study scabies was the only infestation diagnosed.

Pediculosis and tungiasis are still common diseases in Tanzania, but they are mostly considered to be clinically insignificant [6]. No cases of Pediculosis was seen in this study. This could be attributed to patients not seeking medical help or being treated with local traditional methods [10]. Likewise, these skin infestations are treated at primary health care facilities and rarely referred to tertiary hospitals [8, 10].

Eczematous dermatitis, which includes atopic dermatitis, contact dermatitis, seborrheic dermatitis and nummular eczema was the largest group after skin infections. There is a rise in inflammatory condition (28.5 %) compared with 3 % in earlier studies [6]. However, the prevalence of inflammatory conditions are still lower than in developed countries where eczematous dermatitis is more common than skin infections [1, 13]. The findings of this study concurs with a recent study in South Africa [4] that reported a changing trend in skin conditions in the African population.

Pigmentary disorders are generally encountered in older children who are more concerned with their cosmetic appearance [7]. Depigmenting skin conditions are more visible in dark skin. The increase in pigmentary disorders may be due to cosmetic awareness and chronicity of these conditions. Pigmentary diseases were reported to have a negative impact on the patient’s quality of life especially in those with colored skin [14]. Marone et al. [3] in Ethiopia reported that older girls were more cosmetically concerned with changes in their skin color particularly due to vitiligo.

The prevalence of HIV infection among children with skin diseases is 5.8 %. The majority of HIV positive children had inflammatory diseases and Kaposi’s sarcoma. Children living with HIV infection are growing older (mean age 8.4 years) and therefore developing cutaneous manifestations of HIV similar to the adult population. HIV infection is contributing to the changing trends of skin diseases in Africa when compared with studies done before or during the early stages of HIV pandemic.

## Conclusion

Skin infections still remain the leading cause of morbidity among skin diseases despite the urbanization and changing lifestyle in Tanzania although with a reduced frequency. There is an increasing prevalence of inflammatory diseases, pigmentary disorders and malignancies. This changing trends could be attributed to improving socioeconomic status and HIV pandemic.
